# Supplementary material for: Study on the Mechanism of Influencing Adolescents’ Willingness to Participate in Ice Sports
Source: Children (Basel). 2023 Jun 19;10(6):1080. doi: 10.3390/children10061080 (PMC10297628; doi:10.3390/children10061080)
Supplement: Supplementary file 1 [file children-10-01080-s001.zip › children-2388139-supplementary.pdf]

Questionnaire on the mechanisms influencing the willingness of adolescents to participate in ice sports

Hello! I am a current master's student at the School of Physical Education, Shandong University. This survey will provide an understanding of your personal situation and willingness to participate in ice sports for research purposes only. The content of the survey will be kept confidential, so please feel free to fill it out. Thank you for your participation! I wish you progress in your studies and a happy life!

Ⅰ. Basic information section (single choice)

1、Your gender is:

A. Male

B. Female

2、The grade you are studying in is:

A．First year of junior high school

B．Second year of junior high school

C．Third year of junior high school

D．First year of high school

E．Second year of high school

3、Your family's financial situation is:

A．Very well-off

B．Rather well-off

C．Ordinary

D．Difficult

E．Very difficult

II. Survey on Motivation of Adolescents Participation in Ice Sports (Single choice)

1、I want to have a strong and healthy body

A. Strongly agree B. Agree C. Generally D. Disagree E. Strongly disagree

2、I want to lead a healthy life

A. Strongly agree B. Agree C. Generally D. Disagree E. Strongly disagree

3、I want to keep physically and mentally healthy

A. Strongly agree B. Agree C. Generally D. Disagree E. Strongly disagree

4、I want to participate in recreational activities

A. Strongly agree B. Agree C. Generally D. Disagree E. Strongly disagree

5、I want to enjoy a happy life

A. Strongly agree B. Agree C. Generally D. Disagree E. Strongly disagree

6、I want to keep a happy mood

A. Strongly agree B. Agree C. Generally D. Disagree E. Strongly disagree

7、I would like to acquire new motor skills

A. Strongly agree B. Agree C. Generally D. Disagree E. Strongly disagree

8、 I would like to improve my existing motor skills

A. Strongly agree B. Agree C. Generally D. Disagree E. Strongly disagree

9、I would like to maintain my current level of sporting skills

A. Strongly agree B. Agree C. Generally D. Disagree E. Strongly disagree

10、I would like to maintain or improve my figure

A. Strongly agree B. Agree C. Generally D. Disagree E. Strongly disagree

11、I want to control my weight

A. Strongly agree B. Agree C. Generally D. Disagree E. Strongly disagree

12、I would like to make my appearance more attractive

A. Strongly agree B. Agree C. Generally D. Disagree E. Strongly disagree

13、I would like to meet some new friends

A. Strongly agree B. Agree C. Generally D. Disagree E. Strongly disagree

14、I would like to improve my relationship and friendship with my friends

A. Strongly agree B. Agree C. Generally D. Disagree E. Strongly disagree

15、I would like to maintain good social relationships

A. Strongly agree B. Agree C. Generally D. Disagree E. Strongly disagree

III. Survey on Adolescents Participation Opportunities in Ice Sports (Single choice)

1、Would consider participating in ice sports when the sports environment can meet your needs

A. Strongly agree B. Agree C. Generally D. Disagree E. Strongly disagree

2、Would consider participating in ice sports when the sports ground can meet your needs

A. Strongly agree B. Agree C. Generally D. Disagree E. Strongly disagree

3、Would consider participating in ice sports when the sports program can meet your needs

A. Strongly agree B. Agree C. Generally D. Disagree E. Strongly disagree

IV. Survey on Adolescents ' ability to participate in ice sports (single choice)

1、I have knowledge of physical fitness assessment such as body shape and physical ability

A. Strongly agree B. Agree C. Generally D. Disagree E. Strongly disagree

2、I have knowledge of the rules of one or more ice sports

A. Strongly agree B. Agree C. Generally D. Disagree E. Strongly disagree

3、 I have the ability to choose a suitable venue according to the sport of ice

A. Strongly agree B. Agree C. Generally D. Disagree E. Strongly disagree

4、I have the ability to use one or more items of ice sports equipment

A. Strongly agree B. Agree C. Generally D. Disagree E. Strongly disagree

V. Survey on Adolescents Perception of Ice Sports (Single choice)

1、Participating in ice sports is good for me

A. Strongly agree B. Agree C. Generally D. Disagree E. Strongly disagree

2、Participating in ice sports can enrich my social life

A. Strongly agree B. Agree C. Generally D. Disagree E. Strongly disagree

3、Participating in ice sports can help me eliminate negative emotions

A. Strongly agree B. Agree C. Generally D. Disagree E. Strongly disagree

4. I can play ice sports correctly

A. Strongly agree B. Agree C. Generally D. Disagree E. Strongly disagree

5、It is convenient to participate in ice sports

A. Strongly agree B. Agree C. Generally D. Disagree E. Strongly disagree

6、It is not difficult for me to play ice sports

A. Strongly agree B. Agree C. Generally D. Disagree E. Strongly disagree

7、Participating in ice sports may affect safety

A．Strongly agree B．Agree C．Generally D．Disagree E．Strongly disagree

8、Participating in ice sports may cost time or money

A. Strongly agree B. Agree C. Generally D. Disagree E. Strongly disagree

9、Participating in ice sports may be too boring and dull

A. Strongly agree B. Agree C. Generally D. Disagree E. Strongly disagree

VI. Survey on Adolescents ' willingness to participate in snow sports (Single-choice)

1、I am willing to participate in ice sports and related activities

A. Strongly agree B. Agree C. Generally D. Disagree E. Strongly disagree

2、I will insist on participating in ice sports and related activities in the future

A. Strongly agree B. Agree C. Generally D. Disagree E. Strongly disagree

3、On the whole, participating in ice sports has become a habit of my life

A. Strongly agree B. Agree C. Generally D. Disagree E. Strongly disagree

The content of the questionnaire has been completed, thank you for your participation!
